# Supplementary material for: Large language models predict cognition and education close to or better than genomics or expert assessment
Source: Commun Psychol. 2025 Jul 3;3:95. doi: 10.1038/s44271-025-00274-x (PMC12229686; doi:10.1038/s44271-025-00274-x)
Supplement: Supplementary file 2 — Supplementary Information [file 44271_2025_274_MOESM2_ESM.pdf]

# Supplement

# Feature Engineering

## Essays

At age 11, study participants were asked to write an essay whilst being observed by their teachers. The theme of the essay was set as "Imagine you are 25". The exact set of instructions given were as follows:

> *"Imagine you are now 25 years old. Write about the life you are leading, your interests, your home life and your work at the age of 25. You have 30 minutes to do this."*

In total, 10,511 essays of varying length – ranging from 1 to 1,239 words – were transcribed. See Figure S4 for a visualisation of the distribution of essay lengths. We applied various approaches which extract the maximal amount of information from these essays:

- **GPT-based embeddings:** We utilized the `text-embedding-ada-002` model, a pre-trained deep learning natural language processing (NLP) model provided via the OpenAI-Platform API, to generate vector representations of the words in all essays. This model converts each essay into a 1,536-dimensional numeric vector, capturing the semantic meaning of words in their context. These embeddings provide a lower-dimensional representation of the text that preserves important semantic information, allowing for efficient computational analysis of the essays' content using our SuperLearner pipeline.

- **SALAT-metrics:** The Suite of Automatic Linguistic Analysis Tools (SALAT) is a set of open-source linguistic programs. We use it to derive 814 measures related to lexical sophistication, text cohesion, syntactic complexity, lexical diversity, grammar and sentiment for each essay. Keeping only those metrics that are available for all texts, we end up with 534 measures in total.

- **Readability-metrics:** Readability metrics measure the ease with which a reader can understand a written text based on its number of words, sentences, characters, and syllables. The `koRpus`-package provides a set of 31 of such metrics (ARI, Bormuth, Coleman, Coleman.Liau, Dale.Chall, Danielson.Bryan, Dickes.Steiwer, DRP, ELF, Farr.Jenkins.Paterson, Flesch, Flesch.Kincaid, FOG, FORCAST, Fucks, Gutierrez, Harris.Jacobson, Linsear.Write, LIX, nWS, RIX, SMOG, Spache, Strain, Traenkle.Bailer, TRI, Tuldava, Wheeler.Smith), which are computed for every essay.

- **Grammatical and typographical errors:** The number of grammatical and typographical errors in a text carries important information about the capabilities of the author which might be captured neither by the embeddings nor the proposed metrics. We therefore use the LanguageTool CLI via the `LanguageToolR`-package to get a list of more than 184,000 errors in total over all essays which we group into distinct categories like grammar, misspelling, typographical, duplication, and style. For each category, we then calculate error/word-ratios that are used as input to the SuperLearner.

## Teacher Assessments

At age 11, teachers of the respondents were asked to assess their students:

> *"In so far as your professional experience will allow, please rate the child in relation to all children of his age (i.e. not just his present class or even his school) by ringing the number opposite the appropriate description (going from 1 - Exceptional to 5 - Very limited.)"*

For our purposes, four questions are particularly relevant and are used in the analysis:

- General knowledge
- Number work
- Use of books
- Oral ability

As a testament to the teachers' calibration, all four variables exhibit behavior akin to a normal distribution, as can be seen in Figure S5. We extend this selection by six additional items, this time less focused on the scholastic abilities of the child, but more on their behavior and motor abilities:

*"Below are a few descriptions of behavior shown by some children. Any one particular description is likely to apply to only a minority of children; moreover, quite a proportion of children may show at least in some degree one of the aspects of behavior listed below. If the description fits the child, please circle the figure 1 in the first column. If it is a marginal case, or you are in some doubt about the child's inclusion under this description, please circle the figure 2 in the next column. If the description does not fit the child at all circle the figure 3 in the third column (going from 1 - Cert. applies to 3 - Not at all)."*

The list that was provided included the following behavioural descriptors:

- Has poor hand control
- Squirmy, fidgety
- Has poor physical co-ordination
- Hardly ever still
- Has speech difficulties
- Imperfect grasp of English

However, most children are judged to exhibit none of the behaviors in question, as shown in Figure S6. Finally, we utilize an additional set of behavior assessments at age 11 (25), in which a series of behavioral descriptions is given. The teacher is asked to underline the descriptions which best fit the child. By summing the number of selected items, a quantitative assessment of the child's adjustment to school is obtained for multiple categories.

- Inconsequential Behavior
- Nervous Symptoms
- Anxiety for Acceptance (Adults)
- Anxiety for Acceptance (Children)
- Hostility towards Children
- Writing off Adults/Adult Standards
- Hostility towards Adults
- Miscellaneous Symptoms
- Restlessness
- Unforthcomingness
- Depression
- Withdrawal

The maximum score differs for each category. However, most participants received the lowest possible assessment (Figure S7).

## Genomic data

Genetic data for the NCDS cohort were obtained from samples collected during the biomedical sweep conducted between 2002 and 2004. Genotyping was performed on these samples in separate batches using various genotyping chips, followed by independent imputation. The resulting genetic information was then integrated into a comprehensive genetic database, which includes data for 6,435 study participants. A portion of the samples were genotyped as control cases in the WTCCC1, WTCCC2, and T1DGC consortia. Comprehensive details regarding the quality control (QC) and imputation procedures applied to these samples can be found in the works of (55) and (22). In cases where samples were genotyped on multiple chips, preference was given to those that underwent imputation and QC by (55). To maintain consistency, all samples were limited to SNPs in common with the reference panel of the 1,000 Genomes Project. The final sample consisted of 37,772,588 variants on 6,437 individuals. Using PRSice2 (15), we then applied publicly available summary statistics of GWAS to construct polygenic scores for a set of 33 curated traits likely to show associations with the outcomes in question, listed in Table S1. Polygenic scores were calculated using a prespecified threshold of p = 0.5 in the sample and with the software default values for clumping (250kb window; $r^2 = .1$). To assess the robustness of our genetic prediction results, we conducted additional analyses using all polygenic scores made available by the Polygenic Index Repository (7). The Repository PGIs were created using summary statistics from large-scale genome-wide association studies (GWAS), including data from UK Biobank and 23andMe. The PGIs were constructed using Plink2 with SNP weights adjusted for linkage disequilibrium (LD) using LDpred, a method that has been shown to improve the predictive power of polygenic scores compared to traditional methods that do not account for LD. Results for a comparison of both sets of PGSs can be found in Figure S1. The results were largely consistent with our primary findings, Although scores from the PGI-Repository provided slightly less accuracy.

# Outcomes

We seek to evaluate the extent to which genomic data, teacher assessments, and the information embedded in the essays are able to predict a range of outcome variables that can be broadly categorized under three different themes: Cognitive Ability, non-cognitive Traits and Socioeconomic Outcomes.

## Cognitive Ability

An array of tests conducted at age 11 is used to operationalize competencies such as:

- Verbal Ability,
- Nonverbal Ability,
- Reading Ability,
- Mathematical Ability.

The associated variables are given in Table S7. We used these variables to create a measure of general cognitive ability by means of factor analysis. A 1-factor EFA was fitted using the `psych` package (23). All items show high loadings on a general ability factor and together explain more than 75% of the variance. We extract the factor scores from this model as our measure of general intelligence. In addition, two tests of reading comprehension and mathematical ability at age 16 were used. A new reading comprehension test consisted of 35 multiple-choice questions where the child must select the appropriate word to complete a sentence, with scoring based on the number of correct answers. The mathematics test included both numerical and geometric questions with a combination of 27 categorical and four binary questions, scored in a similar manner.

## Non-Cognitive Traits

**Scholastic Motivation**: Scholastic motivation was measured by a battery of self-assessed items in Sweep 3 (age 16) using a five-point Likert-scale expressing agreement with the following statements. The exact wording was:

> *"Below is a list of things that people of your age have said about how they feel towards school. Read each one carefully and then ring one of the numbers to show for each one how true you think what it says is in your own case."*

The associated variables are displayed in Table S8. A one-factor solution once more fits the data well (53.7% variance explained). Note that we use polychoric correlations as we are analyzing Likert-scale data.

**Externalizing Behavior**: A wide range of items at age 16 taken from the Rutter scale was used to construct measures of externalizing behavior (Table S9). Each represents the answer on a scale from one ("Does not apply") to three ("Certainly applies") to the following prompt:

> *"Below is a series of descriptions of behavior often shown by school children. Please ring the appropriate number in each case to show the degree to which the study child exhibits the behavior described. Please complete on the basis of the child's behavior in the past 12 months."*

All items show high loadings on a single factor in an EFA with polychoric correlations whose results were used to generate factor scores of externalizing behavior.

**Internalizing Behaviour**: A further subset of items (Table S10) collected at age 16 was used to construct measures of internalizing behavior. Each one again represents the answer on a scale from one ("Does not apply") to three ("Certainly applies") to the prompt:

> *"Below is a series of descriptions of behavior often shown by school children. Please ring the appropriate number in each case to show the degree to which the study child exhibits the behavior described. Please complete on the basis of the child's behavior in the past 12 months."*

All items show high loadings on a single factor in an EFA with polychoric correlations whose results were used to generate factor scores of internalizing behavior.

**Occupational Aspirations**: At age 16, cohort members were asked about the first job they aspired to have (variable name: n2771). The distribution is displayed in Figure S8. These occupations were mapped to the 1970 Classification of Occupations (variable name: co70) which was the current statistical standard in 1974 when the cohort members were interviewed. Using the mapping to co70 provided by the CAMSIS-project (16), a measure of occupational status was then assigned to each occupation. Note that different occupations are mapped to different CAMSIS scores depending on whether the respondent is an employer, employee, or self-employed. As this information was not available for the occupation in question, the score for employee was used.

## Educational Attainment

A measure of the highest education obtained up to age 33 is available in Sweep 5 (variable name: hqual33) as a six-level outcome, ranging from *'No qualification'* to *'Degree/higher NVQ 5,6'*. It was treated as an ordinal variable in all analyses.

# Additional Analyses

## Incremental Utility of SuperLearner over OLS

We investigated to what extent the utility of the SuperLearner-based ensemble of machine learning methods used in this study exceeds standard OLS regressions. For this purpose, we ran 10-fold cross-validated OLS regressions using our three sets of covariates (teacher assessments, essays, and genetic data) in order to predict cognitive and non-cognitive outcomes. We then computed the difference in $R^2_{\text{Holdout}}$ to the respective SuperLearner model. The comparison is plotted in Figure S9. We note virtually identical results for teacher assessments and genetic data, and improvements for the essay-based predictors. For multiple non-cognitive outcomes, OLS-based prediction under-performs a simple model using only the mean of the dependent variable in the training sample, leading to negative $R^2_{\text{Holdout}}$ values. In these cases, the difference to zero was calculated.

## Predicting Personality at Age 50

To extend the set of non-cognitive traits in our analysis, we also tested if essays, polygenic scores, and teacher evaluations were able to predict dimensions of personality. Unfortunately, the earliest measure of personality is available at age 50: The NCDS self-completion questionnaire in Sweep 8 contained 50 questions from the International Personality Item Pool (IPIP, 9), as shown in Table S11. The answers can be summed to obtain scores for the 'Big-5' personality traits: extraversion, neuroticism, conscientiousness, agreeableness, and openness to experience. The scores for each trait range from 5 to 50, with higher scores corresponding to a higher expression of the respective trait. Even though our predictors are fixed at birth (genetic markers) or at age 11 (essays and teacher assessments) and the outcome is measured later in life (educational attainment at age 33), the mean predictive $R^2$ over all folds is greater than zero for all traits. This is highest for agreeableness and openness, where more than 10% of the variance can be explained by the essays (Figure S10).

## Robustness of Results Regarding Sample Composition

As our analyses depend on variables with different patterns of nonresponse, we use all available observations in each of our models, leading to differences in sample composition that might influence results, as shown in Table S12. To show that sample composition does not affect our findings, we reran our analysis on the subset of individuals with full information on every variable used in the main text, meaning genotyped respondents with available essays, teacher assessments, (non-)cognitive information, highest education at age 33, parental education, height at age 11, sex, and birthweight. This final set consisted of only 1,618 individuals. Results are displayed in Figure S11. In this case, variation in predictive performance over all cross-validation folds is much larger compared to our complete-case models, a sensible finding as each fold consists of fewer observations (and 'learning error' might not be fully eliminated). However, in all cases, the intervals denoting the range from worst to best prediction fold clearly overlap. The same holds true for our prediction of educational attainment, both for the stepwise prediction using combinations of teacher assessments, polygenic scores, and essays (Figure S12), where the results of different folds clearly overlap.

# Supplemental References


[1] Barban, N., R. Jansen, R. de Vlaming, A. Vaez, J. J. Mandemakers, F. C. Tropf, X. Shen, J. F. Wilson, D. I. Chasman, I. M. Nolte, V. Tragante, S. W. van der Laan, J. R. B. Perry, A. Kong, T. S. Ahluwalia, E. Albrecht, L. Yerges-Armstrong, G. Atzmon, K. Auro, K. Ayers, A. Bakshi, D. Ben-Avraham, K. Berger, A. Bergman, L. Bertram, L. F. Bielak, G. Bjornsdottir, M. J. Bonder, L. Broer, M. Bui, C. Barbieri, A. Cavadino, J. E. Chavarro, C. Turman, M. P. Concas, H. J. Cordell, G. Davies, P. Eibich, N. Eriksson, T. Esko, J. Eriksson, F. Falahi, J. F. Felix, M. A. Fontana, L. Franke, I. Gandin, A. J. Gaskins, C. Gieger, E. P. Gunderson, X. Guo, C. Hayward, C. He, E. Hofer, H. Huang, P. K. Joshi, S. Kanoni, R. Karlsson, S. Kiechl, A. Kifley, A. Kluttig, P. Kraft, V. Lagou, C. Lecoeur, J. Lahti, R. Li-Gao, P. A. Lind, T. Liu, E. Makalic, C. Mamasoula, L. Matteson, H. Mbarek, P. F. McArdle, G. McMahon, S. F. W. Meddens, E. Mihailov, M. Miller, S. A. Missmer, C. Monnereau, P. J. van der Most, R. Myhre, M. A. Nalls, T. Nutile, I. P. Kalafati, E. Porcu, I. Prokopenko, K. B. Rajan, J. Rich-Edwards, C. A. Rietveld, A. Robino, L. M. Rose, R. Rueedi, K. A. Ryan, Y. Saba, D. Schmidt, J. A. Smith, L. Stolk, E. Streeten, A. Tönjes, G. Thorleifsson, S. Ulivi, J. Wedenoja, J. Wellmann, P. Willeit, J. Yao, L. Yengo, J. H. Zhao, W. Zhao, D. V. Zhernakova, N. Amin, H. Andrews, B. Balkau, N. Barzilai, S. Bergmann, G. Biino, H. Bisgaard, K. Bønnelykke, D. I. Boomsma, J. E. Buring, H. Campbell, S. Cappellani, M. Ciullo, S. R. Cox, F. Cucca, D. Toniolo, G. Davey-Smith, I. J. Deary, G. Dedoussis, P. Deloukas, C. M. van Duijn, E. J. C. de Geus, J. G. Eriksson, D. A. Evans, J. D. Faul, C. F. Sala, P. Froguel, P. Gasparini, G. Girotto, H.-J. Grabe, K. H. Greiser, P. J. F. Groenen, H. G. de Haan, J. Haerting, T. B. Harris, A. C. Heath, K. Heikkilä, A. Hofman, G. Homuth, E. G. Holliday, J. Hopper, E. Hyppönen, B. Jacobsson, V. W. V. Jaddoe, M. Johannesson, A. Jugessur, M. Kähönen, E. Kajantie, S. L. R. Kardia, B. Keavney, I. Kolcic, P. Koponen, P. Kovacs, F. Kronenberg, Z. Kutalik, M. La Bianca, G. Lachance, W. G. Iacono, S. Lai, T. Lehtimäki, D. C. Liewald, C. M. Lindgren, Y. Liu, R. Luben, M. Lucht, R. Luoto, P. Magnus, P. K. E. Magnusson, N. G. Martin, M. McGue, R. McQuillan, S. E. Medland, C. Meisinger, D. Mellström, A. Metspalu, M. Traglia, L. Milani, P. Mitchell, G. W. Montgomery, D. Mook-Kanamori, R. de Mutsert, E. A. Nohr, C. Ohlsson, J. Olsen, K. K. Ong, L. Paternoster, A. Pattie, B. W. J. H. Penninx, M. Perola, P. A. Peyser, M. Pirastu, O. Polasek, C. Power, J. Kaprio, L. J. Raffel, K. Räikkönen, O. Raitakari, P. M. Ridker, S. M. Ring, K. Roll, I. Rudan, D. Ruggiero, D. Rujescu, V. Salomaa, D. Schlessinger, H. Schmidt, R. Schmidt, N. Schupf, J. Smit, R. Sorice, T. D. Spector, J. M. Starr, D. Stöckl, K. Strauch, M. Stumvoll, M. A. Swertz, U. Thorsteinsdottir, A. R. Thurik, N. J. Timpson, J. Y. Tung, A. G. Uitterlinden, S. Vaccargiu, J. Viikari, V. Vitart, H. Völzke, P. Vollenweider, D. Vuckovic, J. Waage, G. G. Wagner, J. J. Wang, N. J. Wareham, D. R. Weir, G. Willemsen, J. Willeit, A. F. Wright, K. T. Zondervan, K. Stefansson, R. F. Krueger, J. J. Lee, D. J. Benjamin, D. Cesarini, P. D. Koellinger, M. den Hoed, H. Snieder, and M. C. Mills (2016, December). Genome-wide analysis identifies 12 loci influencing human reproductive behavior. *Nature Genetics 48*(12), 1462–1472.

[2] Baselmans, B. M. L., R. Jansen, H. F. Ip, J. van Dongen, A. Abdellaoui, M. P. van de Weijer, Y. Bao, M. Smart, M. Kumari, G. Willemsen, J.-J. Hottenga, D. I. Boomsma, E. J. C. de Geus, M. G. Nivard, and M. Bartels (2019, March). Multivariate genome-wide analyses of the well-being spectrum. *Nature Genetics 51*(3), 445–451.

[7] Becker, J., C. A. Burik, G. Goldman, N. Wang, H. Jayashankar, M. Bennett, D. W. Belsky, R. Karlsson Linnér, R. Ahlskog, A. Kleinman, et al. (2021). Resource profile and user guide of the polygenic index repository. *Nature human behaviour 5*(12), 1744–1758.

[15] Choi, S. W. and P. F. O'Reilly (2019). PRSice-2: Polygenic Risk Score software for biobank-scale data. *Gigascience 8*(7), giz082.

[5] Day, F. R., K. K. Ong, and J. R. B. Perry (2018, July). Elucidating the genetic basis of social interaction and isolation. *Nature Communications 9*(1), 2457.

[6] de la Fuente, J., G. Davies, A. D. Grotzinger, E. M. Tucker-Drob, and I. J. Deary (2021). A general dimension of genetic sharing across diverse cognitive traits inferred from molecular data. *Nature Human Behaviour 5*(1), 49–58.

[7] Demange, P. A., M. Malanchini, T. T. Mallard, P. Biroli, S. R. Cox, A. D. Grotzinger, E. M. Tucker-Drob, A. Abdellaoui, L. Arseneault, E. van Bergen, D. I. Boomsma, A. Caspi, D. L. Corcoran, B. W. Domingue, K. M. Harris, H. F. Ip, C. Mitchell, T. E. Moffitt, R. Poulton, J. A. Prinz, K. Sugden, J. Wertz, B. S. Williams, E. L. de Zeeuw, D. W. Belsky, K. P. Harden, and M. G. Nivard (2021, January). Investigating the genetic architecture of noncognitive skills using GWAS-by-subtraction. *Nature Genetics 53*(1), 35–44.

[8] Demontis, D., R. K. Walters, J. Martin, M. Mattheisen, T. D. Als, E. Agerbo, G. Baldursson, R. Belliveau, J. Bybjerg-Grauholm, M. Bækvad-Hansen, et al. (2019). Discovery of the first genome-wide significant risk loci for attention deficit/hyperactivity disorder. *Nature genetics 51*(1), 63–75.

[9] Goldberg, L. R., J. A. Johnson, H. W. Eber, R. Hogan, M. C. Ashton, C. R. Cloninger, and H. G. Gough (2006). The international personality item pool and the future of public-domain personality measures. *Journal of Research in personality 40*(1), 84–96.

[10] Grove, J., S. Ripke, T. D. Als, M. Mattheisen, R. K. Walters, H. Won, J. Pallesen, E. Agerbo, O. A. Andreassen, R. Anney, S. Awashti, R. Belliveau, F. Bettella, J. D. Buxbaum, J. Bybjerg-Grauholm, M. Bækvad-Hansen, F. Cerrato, K. Chambert, J. H. Christensen, C. Churchhouse, K. Dellenvall, D. Demontis, S. De Rubeis, B. Devlin, S. Djurovic, A. L. Dumont, J. I. Goldstein, C. S. Hansen, M. E. Hauberg, M. V. Hollegaard, S. Hope, D. P. Howrigan, H. Huang, C. M. Hultman, L. Klei, J. Maller, J. Martin, A. R. Martin, J. L. Moran, M. Nyegaard, T. Nærland, D. S. Palmer, A. Palotie, C. B. Pedersen, M. G. Pedersen, T. dPoterba, J. B. Poulsen, B. S. Pourcain, P. Qvist, K. Rehnström, A. Reichenberg, J. Reichert, E. B. Robinson, K. Roeder, P. Roussos, E. Saemundsen, S. Sandin, F. K. Satterstrom, G. Davey Smith, H. Stefansson, S. Steinberg, C. R. Stevens, P. F. Sullivan, P. Turley, G. B. Walters, X. Xu, Autism Spectrum Disorder Working Group of the Psychiatric Genomics Consortium, BUPGEN, Major Depressive Disorder Working Group of the Psychiatric Genomics Consortium, 23andMe Research Team, K. Stefansson, D. H. Geschwind, M. Nordentoft, D. M. Hougaard, T. Werge, O. Mors, P. B. Mortensen, B. M. Neale, M. J. Daly, and A. D. Børglum (2019, March). Identification of common genetic risk variants for autism spectrum disorder. *Nature Genetics 51*(3), 431–444.

[11] Hill, D., N. M. Davies, S. J. Ritchie, N. G. Skene, J. Bryois, S. Bell, E. Di Angelantonio, D. J. Roberts, S. Xueyi, G. Davies, D. C. M. Liewald, D. J. Porteous, C. Hayward, A. S. Butterworth, A. M. McIntosh, C. R. Gale, and I. J. Deary (2019, December). Genome-wide analysis identifies molecular systems and 149 genetic loci associated with income. *Nature Communications 10*(1), 5741.

[12] Hill, W. D., A. Weiss, D. C. Liewald, G. Davies, D. J. Porteous, C. Hayward, A. M. McIntosh, C. R. Gale, and I. J. Deary (2020, November). Genetic contributions to two special factors of neuroticism are associated with affluence, higher intelligence, better health, and longer life. *Molecular Psychiatry 25*(11), 3034–3052.

[13] Howard, D. M., M. J. Adams, T.-K. Clarke, J. D. Hafferty, J. Gibson, M. Shirali, J. R. I. Coleman, S. P. Hagenaars, J. Ward, E. M. Wigmore, C. Alloza, X. Shen, M. C. Barbu, E. Y. Xu, H. C. Whalley, R. E. Marioni, D. J. Porteous, G. Davies, I. J. Deary, G. Hemani, K. Berger, H. Teismann, R. Rawal, V. Arolt, B. T. Baune, U. Dannlowski, K. Domschke, C. Tian, D. A. Hinds, M. Trzaskowski, E. M. Byrne, S. Ripke, D. J. Smith, P. F. Sullivan, N. R. Wray, G. Breen, C. M. Lewis, and A. M. McIntosh (2019, March). Genome-wide meta-analysis of depression identifies 102 independent variants and highlights the importance of the prefrontal brain regions. *Nature neuroscience 22*(3), 343–352.

[14] Johnson, E. C., D. Demontis, T. E. Thorgeirsson, R. K. Walters, R. Polimanti, A. S. Hatoum, S. Sanchez-Roige, S. E. Paul, F. R. Wendt, T.-K. Clarke, D. Lai, G. W. Reginsson, H. Zhou, J. He, D. A. A. Baranger, D. F. Gudbjartsson, R. Wedow, D. E. Adkins, A. E. Adkins, J. Alexander, S.-A. Bacanu, T. B. Bigdeli, J. Boden, S. A. Brown, K. K. Bucholz, J. Bybjerg-Grauholm, R. P. Corley, L. Degenhardt, D. M. Dick, B. W. Domingue, L. Fox, A. M. Goate, S. D. Gordon, L. M. Hack, D. B. Hancock, S. M. Hartz, I. B. Hickie, D. M. Hougaard, K. Krauter, P. A. Lind, J. N. McClintick, M. B. McQueen, J. L. Meyers, G. W. Montgomery, O. Mors, P. B. Mortensen, M. Nordentoft, J. F. Pearson, R. E. Peterson, M. D. Reynolds, J. P. Rice, V. Runarsdottir, N. L. Saccone, R. Sherva, J. L. Silberg, R. E. Tarter, T. Tyrfingsson, T. L. Wall, B. T. Webb, T. Werge, L. Wetherill, M. J. Wright, S. Zellers, M. J. Adams, L. J. Bierut, J. D. Boardman, W. E. Copeland, L. A. Farrer, T. M. Foroud, N. A. Gillespie, R. A. Grucza, K. M. Harris, A. C. Heath, V. Hesselbrock, J. K. Hewitt, C. J. Hopfer, J. Horwood, W. G. Iacono, E. O. Johnson, K. S. Kendler, M. A. Kennedy, H. R. Kranzler, P. A. F. Madden, H. H. Maes, B. S. Maher, N. G. Martin, M. McGue, A. M. McIntosh, S. E. Medland, E. C. Nelson, B. Porjesz, B. P. Riley, M. C. Stallings, M. M. Vanyukov, S. Vrieze, R. Walters, R. Polimanti, E. Johnson, J. McClintick, A. Hatoum, J. He, F. Wendt, H. Zhou, M. Adams, A. Adkins, F. Aliev, S.-A. Bacanu, A. Batzler, S. Bertelsen, J. Biernacka, T. Bigdeli, L.-S. Chen, T.-K. Clarke, Y.-L. Chou, F. Degenhardt, A. Docherty, A. Edwards, P. Fontanillas, J. Foo, L. Fox, J. Frank, I. Giegling, S. Gordon, L. Hack, A. Hartmann, S. Hartz, S. Heilmann-Heimbach, S. Herms, C. Hodgkinson, P. Hoffman, J. Hottenga, M. Kennedy, M. Alanne-Kinnunen, B. Konte, J. Lahti, M. Lahti-Pulkkinen, D. Lai, L. Ligthart, A. Loukola, B. Maher, H. Mbarek, A. McIntosh, M. McQueen, J. Meyers, Y. Milaneschi, T. Palviainen, J. Pearson, R. Peterson, S. Ripatti, E. Ryu, N. Saccone, J. Salvatore, S. Sanchez-Roige, M. Schwandt, R. Sherva, F. Streit, J. Strohmaier, N. Thomas, J.-C. Wang, B. Webb, R. Wedow, L. Wetherill, A. Wills, J. Boardman, D. Chen, D.-S. Choi, W. Copeland, R. Culverhouse, N. Dahmen, L. Degenhardt, B. Domingue, S. Elson, M. Frye, W. Gäbel, C. Hayward, M. Ising, M. Keyes, F. Kiefer, J. Kramer, S. Kuperman, S. Lucae, M. Lynskey, W. Maier, K. Mann, S. Männistö, B. Müller-Myhsok, A. Murray, J. Nurnberger, A. Palotie, U. Preuss, K. Räikkönen, M. Reynolds, M. Ridinger, N. Scherbaum, M. Schuckit, M. Soyka, J. Treutlein, S. Witt, N. Wodarz, P. Zill, D. Adkins, J. Boden, D. Boomsma, L. Bierut, S. Brown, K. Bucholz, S. Cichon, E. J. Costello, H. de Wit, N. Diazgranados, D. Dick, J. Eriksson, L. Farrer, T. Foroud, N. Gillespie, A. Goate, D. Goldman, R. Grucza, D. Hancock, K. M. Harris, A. Heath, V. Hesselbrock, J. Hewitt, C. Hopfer, J. Horwood, W. Iacono, E. Johnson, J. Kaprio, V. Karpyak, K. Kendler, H. Kranzler, K. Krauter, P. Lichtenstein, P. Lind, M. McGue, J. MacKillop,

P. Madden, H. Maes, P. Magnusson, N. Martin, S. Medland, G. Montgomery, E. Nelson, M. Nöthen, A. Palmer, N. Pederson, B. Penninx, B. Porjesz, J. Rice, M. Rietschel, B. Riley, R. Rose, D. Rujescu, P.-H. Shen, J. Silberg, M. Stallings, R. Tarter, M. Vanyukov, S. Vrieze, T. Wall, J. Whitfield, H. Zhao, B. Neale, J. Gelernter, H. Edenberg, A. Agrawal, L. K. Davis, R. Bogdan, J. Gelernter, H. J. Edenberg, K. Stefansson, A. D. Børglum, and A. Agrawal (2020, December). A large-scale genome-wide association study meta-analysis of cannabis use disorder. *The Lancet Psychiatry 7*(12), 1032–1045.

[15] Karlsson Linnér, R., P. Biroli, E. Kong, S. F. W. Meddens, R. Wedow, M. A. Fontana, M. Lebreton, S. P. Tino, A. Abdellaoui, A. R. Hammerschlag, M. G. Nivard, A. Okbay, C. A. Rietveld, P. N. Timshel, M. Trzaskowski, R. de Vlaming, C. L. Zünd, Y. Bao, L. Buzdugan, A. H. Caplin, C.-Y. Chen, P. Eibich, P. Fontanillas, J. R. Gonzalez, P. K. Joshi, V. Karhunen, A. Kleinman, R. Z. Levin, C. M. Lill, G. A. Meddens, G. Muntané, S. Sanchez-Roige, F. J. van Rooij, E. Taskesen, Y. Wu, F. Zhang, A. Auton, J. D. Boardman, D. W. Clark, A. Conlin, C. C. Dolan, U. Fischbacher, P. J. F. Groenen, K. M. Harris, G. Hasler, A. Hofman, M. A. Ikram, S. Jain, R. Karlsson, R. C. Kessler, M. Kooyman, J. MacKillop, M. Männikkö, C. Morcillo-Suarez, M. B. McQueen, K. M. Schmidt, M. C. Smart, M. Sutter, A. R. Thurik, A. G. Uitterlinden, J. White, H. de Wit, J. Yang, L. Bertram, M. Jan Bonder, D. I. Boomsma, T. Esko, E. Fehr, D. A. Hinds, M. Johannesson, M. Kumari, D. Laibson, P. K. E. Magnusson, M. N. Meyer, A. Navarro, A. A. Palmer, T. H. Pers, D. Posthuma, D. Schunk, M. B. Stein, R. Svento, H. Tiemeier, P. R. H. J. Timmers, P. Turley, R. J. Ursano, G. G. Wagner, J. F. Wilson, J. Gratten, Z. Kutalik, J. J. Lee, D. Cesarini, D. J. Benjamin, P. D. Koellinger, and J. P. Beauchamp (2019, February). Genome-wide association analyses of risk tolerance and risky behaviors in over 1 million individuals identify hundreds of loci and shared genetic influences. *Nature Genetics 51*(2), 245–257.

[16] Lambert, P. and K. Prandy (2023). Camsis project webpages: Cambridge social interaction and stratification scales. *Last Accessed: 06/08/2023.*

[38] Lee, J. J., R. Wedow, A. Okbay, E. Kong, O. Maghzian, M. Zacher, T. A. Nguyen-Viet, P. Bowers, J. Sidorenko, R. K. Linnér, M. A. Fontana, T. Kundu, C. Lee, H. Li, R. Li, R. Royer, P. N. Timshel, R. K. Walters, E. A. Willoughby, L. Yengo, M. Alver, Y. Bao, D. W. Clark, F. R. Day, N. A. Furlotte, P. K. Joshi, K. E. Kemper, A. Kleinman, C. Langenberg, R. Mägi, J. W. Trampush, S. S. Verma, Y. Wu, M. Lam, J. H. Zhao, Z. Zheng, J. D. Boardman, H. Campbell, J. Freese, K. M. Harris, C. Hayward, P. Herd, M. Kumari, T. Lencz, J. Luan, A. K. Malhotra, A. Metspalu, L. Milani, K. K. Ong, J. R. B. Perry, D. J. Porteous, M. D. Ritchie, M. C. Smart, B. H. Smith, J. Y. Tung, N. J. Wareham, J. F. Wilson, J. P. Beauchamp, D. C. Conley, T. Esko, S. F. Lehrer, P. K. E. Magnusson, S. Oskarsson, T. H. Pers, M. R. Robinson, K. Thom, C. Watson, C. F. Chabris, M. N. Meyer, D. I. Laibson, J. Yang, M. Johannesson, P. D. Koellinger, P. Turley, P. M. Visscher, D. J. Benjamin, and D. Cesarini (2018, August). Gene discovery and polygenic prediction from a genome-wide association study of educational attainment in 1.1 million individuals. *Nature Genetics 50*(8), 1112–1121.

[18] Liu, M., Y. Jiang, R. Wedow, Y. Li, D. M. Brazel, F. Chen, G. Datta, J. Davila-Velderrain, D. McGuire, C. Tian, X. Zhan, H. Choquet, A. R. Docherty, J. D. Faul, J. R. Foerster, L. G. Fritsche, M. E. Gabrielsen, S. D. Gordon, J. Haessler, J.-J. Hottenga, H. Huang, S.-K. Jang, P. R. Jansen, Y. Ling, R. Mägi, N. Matoba, G. McMahon, A. Mulas, V. Orrù, T. Palviainen, A. Pandit, G. W. Reginsson, A. H. Skogholt, J. A. Smith, A. E. Taylor, C. Turman, G. Willemsen, H. Young, K. A. Young, G. J. M. Zajac, W. Zhao, W. Zhou, G. Bjornsdottir, J. D. Boardman, M. Boehnke, D. I. Boomsma, C. Chen, F. Cucca, G. E. Davies, C. B. Eaton, M. A. Ehringer, T. Esko, E. Fiorillo, N. A. Gillespie, D. F. Gudbjartsson, T. Haller, K. M. Harris, A. C. Heath, J. K. Hewitt, I. B. Hickie, J. E. Hokanson, C. J. Hopfer, D. J. Hunter, W. G. Iacono, E. O. Johnson, Y. Kamatani, S. L. R. Kardia, M. C. Keller, M. Kellis, C. Kooperberg, P. Kraft, K. S. Krauter, M. Laakso, P. A. Lind, A. Loukola, S. M. Lutz, P. A. F. Madden, N. G. Martin, M. McGue, M. B. McQueen, S. E. Medland, A. Metspalu, K. L. Mohlke, J. B. Nielsen, Y. Okada, U. Peters, T. J. C. Polderman, D. Posthuma, A. P. Reiner, J. P. Rice, E. Rimm, R. J. Rose, V. Runarsdottir, M. C. Stallings, A. Stančáková, H. Stefansson, K. K. Thai, H. A. Tindle, T. Tyrfingsson, T. L. Wall, D. R. Weir, C. Weisner, J. B. Whitfield, B. S. Winsvold, J. Yin, L. Zuccolo, L. J. Bierut, K. Hveem, J. J. Lee, M. R. Munafò, N. L. Saccone, C. J. Willer, M. C. Cornelis, S. P. David, D. A. Hinds, E. Jorgenson, J. Kaprio, J. A. Stitzel, K. Stefansson, T. E. Thorgeirsson, G. Abecasis, D. J. Liu, and S. Vrieze (2019, February). Association studies of up to 1.2 million individuals yield new insights into the genetic etiology of tobacco and alcohol use. *Nature Genetics 51*(2), 237–244.

[19] Lo, M.-T., D. A. Hinds, J. Y. Tung, C. Franz, C.-C. Fan, Y. Wang, O. B. Smeland, A. Schork, D. Holland, K. Kauppi, N. Sanyal, V. Escott-Price, D. J. Smith, M. O'Donovan, H. Stefansson, G. Bjornsdottir, T. E. Thorgeirsson, K. Stefansson, L. K. McEvoy, A. M. Dale, O. A. Andreassen, and C.-H. Chen (2017, January). Genome-wide analyses for personality traits identify six genomic loci and show correlations with psychiatric disorders. *Nature Genetics 49*(1), 152–156.

[20] Mullins, N., A. J. Forstner, K. S. O'Connell, B. Coombes, J. R. I. Coleman, Z. Qiao, T. D. Als, T. B. Bigdeli, S. Børte, J. Bryois, A. W. Charney, O. K. Drange, M. J. Gandal, S. P. Hagenaars, M. Ikeda, N. Kamitaki,

M. Kim, K. Krebs, G. Panagiotaropoulou, B. M. Schilder, L. G. Sloofman, S. Steinberg, V. Trubetskoy, B. S. Winsvold, H.-H. Won, L. Abramova, K. Adorjan, E. Agerbo, M. Al Eissa, D. Albani, N. Alliey-Rodriguez, A. Anjorin, V. Antilla, A. Antoniou, S. Awasthi, J. H. Baek, M. Bækvad-Hansen, N. Bass, M. Bauer, E. C. Beins, S. E. Bergen, A. Birner, C. Bøcker Pedersen, E. Bøen, M. P. Boks, R. Bosch, M. Brum, B. M. Brumpton, N. Brunkhorst-Kanaan, M. Budde, J. Bybjerg-Grauholm, W. Byerley, M. Cairns, M. Casas, P. Cervantes, T.-K. Clarke, C. Cruceanu, A. Cuellar-Barboza, J. Cunningham, D. Curtis, P. M. Czerski, A. M. Dale, N. Dalkner, F. S. David, F. Degenhardt, S. Djurovic, A. L. Dobbyn, A. Douzenis, T. Elvsåshagen, V. Escott-Price, I. N. Ferrier, A. Fiorentino, T. M. Foroud, L. Forty, J. Frank, O. Frei, N. B. Freimer, L. Frisén, K. Gade, J. Garnham, J. Gelernter, M. Giørtz Pedersen, I. R. Gizer, S. D. Gordon, K. Gordon-Smith, T. A. Greenwood, J. Grove, J. Guzman-Parra, K. Ha, M. Haraldsson, M. Hautzinger, U. Heilbronner, D. Hellgren, S. Herms, P. Hoffmann, P. A. Holmans, L. Huckins, S. Jamain, J. S. Johnson, J. L. Kalman, Y. Kamatani, J. L. Kennedy, S. Kittel-Schneider, J. A. Knowles, M. Kogevinas, M. Koromina, T. M. Kranz, H. R. Kranzler, M. Kubo, R. Kupka, S. A. Kushner, C. Lavebratt, J. Lawrence, M. Leber, H.-J. Lee, P. H. Lee, S. E. Levy, C. Lewis, C. Liao, S. Lucae, M. Lundberg, D. J. MacIntyre, S. H. Magnusson, W. Maier, A. Maihofer, D. Malaspina, E. Maratou, L. Martinsson, M. Mattheisen, S. A. McCarroll, N. W. McGregor, P. McGuffin, J. D. McKay, H. Medeiros, S. E. Medland, V. Millischer, G. W. Montgomery, J. L. Moran, D. W. Morris, T. W. Mühleisen, N. O'Brien, C. O'Donovan, L. M. Olde Loohuis, L. Oruc, S. Papiol, A. F. Pardiñas, A. Perry, A. Pfennig, E. Porichi, J. B. Potash, D. Quested, T. Raj, M. H. Rapaport, J. R. DePaulo, E. J. Regeer, J. P. Rice, F. Rivas, M. Rivera, J. Roth, P. Roussos, D. M. Ruderfer, C. Sánchez-Mora, E. C. Schulte, F. Senner, S. Sharp, P. D. Shilling, E. Sigurdsson, L. Sirignano, C. Slaney, O. B. Smeland, D. J. Smith, J. L. Sobell, C. Søholm Hansen, M. Soler Artigas, A. T. Spijker, D. J. Stein, J. S. Strauss, B. Świątkowska, C. Terao, T. E. Thorgeirsson, C. Toma, P. Tooney, E.-E. Tsermpini, M. P. Vawter, H. Vedder, J. T. R. Walters, S. H. Witt, S. Xi, W. Xu, J. M. K. Yang, A. H. Young, H. Young, P. P. Zandi, H. Zhou, L. Zillich, R. Adolfsson, I. Agartz, M. Alda, L. Alfredsson, G. Babadjanova, L. Backlund, B. T. Baune, F. Bellivier, S. Bengesser, W. H. Berrettini, D. H. R. Blackwood, M. Boehnke, A. D. Børglum, G. Breen, V. J. Carr, S. Catts, A. Corvin, N. Craddock, U. Dannlowski, D. Dikeos, T. Esko, B. Etain, P. Ferentinos, M. Frye, J. M. Fullerton, M. Gawlik, E. S. Gershon, F. S. Goes, M. J. Green, M. Grigoroiu-Serbanescu, J. Hauser, F. Henskens, J. Hillert, K. S. Hong, D. M. Hougaard, C. M. Hultman, K. Hveem, N. Iwata, A. V. Jablensky, I. Jones, L. A. Jones, R. S. Kahn, J. R. Kelsoe, G. Kirov, M. Landén, M. Leboyer, C. M. Lewis, Q. S. Li, J. Lissowska, C. Lochner, C. Loughland, N. G. Martin, C. A. Mathews, F. Mayoral, S. L. McElroy, A. M. McIntosh, F. J. McMahon, I. Melle, P. Michie, L. Milani, P. B. Mitchell, G. Morken, O. Mors, P. B. Mortensen, B. Mowry, B. Müller-Myhsok, R. M. Myers, B. M. Neale, C. M. Nievergelt, M. Nordentoft, M. M. Nöthen, M. C. O'Donovan, K. J. Oedegaard, T. Olsson, M. J. Owen, S. A. Paciga, C. Pantelis, C. Pato, M. T. Pato, G. P. Patrinos, R. H. Perlis, D. Posthuma, J. A. Ramos-Quiroga, A. Reif, E. Z. Reininghaus, M. Ribasés, M. Rietschel, S. Ripke, G. A. Rouleau, T. Saito, U. Schall, M. Schalling, P. R. Schofield, T. G. Schulze, L. J. Scott, R. J. Scott, A. Serretti, C. Shannon Weickert, J. W. Smoller, H. Stefansson, K. Stefansson, E. Stordal, F. Streit, P. F. Sullivan, G. Turecki, A. E. Vaaler, E. Vieta, J. B. Vincent, I. D. Waldman, T. W. Weickert, T. Werge, N. R. Wray, J.-A. Zwart, J. M. Biernacka, J. I. Nurnberger, S. Cichon, H. J. Edenberg, E. A. Stahl, A. McQuillin, A. Di Florio, R. A. Ophoff, and O. A. Andreassen (2021, June). Genome-wide association study of more than 40,000 bipolar disorder cases provides new insights into the underlying biology. *Nature Genetics 53*(6), 817–829.

[42] Plomin, R. (2018). *Blueprint: How DNA Makes Us Who We Are.* Mit Press.

[22] Psychiatric Genomics Consortium (2014, July). Biological insights from 108 schizophrenia-associated genetic loci. *Nature 511*(7510), 421–427.

[23] Revelle, W. R. (2017). Psych: Procedures for personality and psychological research.

[24] Sanchez-Roige, S., A. A. Palmer, P. Fontanillas, S. L. Elson, 23andMe Research Team, the Substance Use Disorder Working Group of the Psychiatric Genomics Consortium, M. J. Adams, D. M. Howard, H. J. Edenberg, G. Davies, R. C. Crist, I. J. Deary, A. M. McIntosh, and T.-K. Clarke (2019, February). Genome-Wide Association Study Meta-Analysis of the Alcohol Use Disorders Identification Test (AUDIT) in Two Population-Based Cohorts. *The American Journal of Psychiatry 176*(2), 107–118.

[25] Shepherd, P. (2013). Bristol social adjustment guides at 7 and 11 years. *Centre for Longitudinal Studies.*

[26] Watanabe, K., S. Stringer, O. Frei, M. Umićević Mirkov, C. de Leeuw, T. J. C. Polderman, S. van der Sluis, O. A. Andreassen, B. M. Neale, and D. Posthuma (2019, September). A global overview of pleiotropy and genetic architecture in complex traits. *Nature Genetics 51*(9), 1339–1348.

# Supplementary Figures

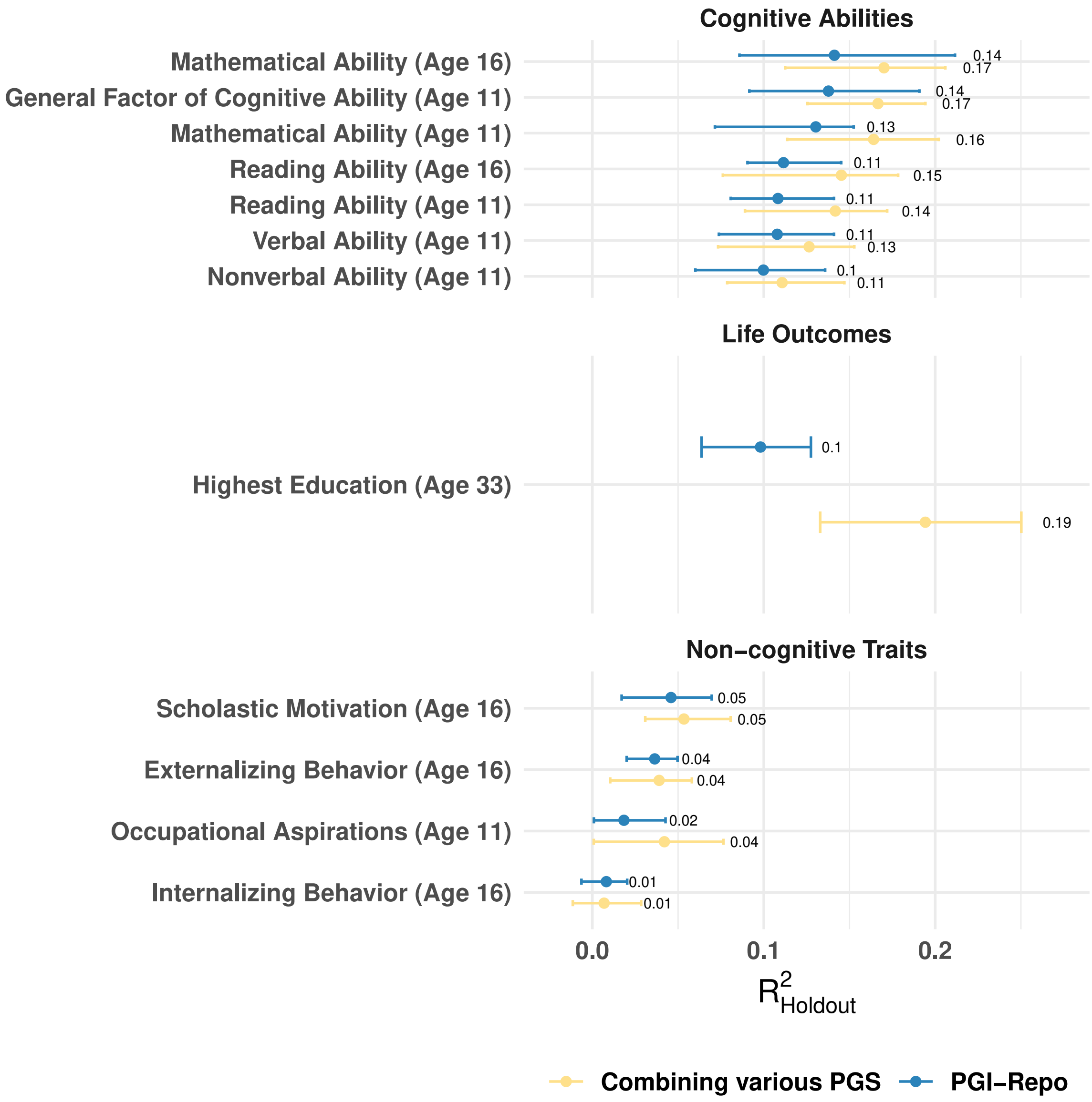


**Figure S1:** Comparison of the predictive power of various combined PGSs as created by our algorithmic pipeline (light blue), and various PGSs as provided by the Polygenic Index (PGI) Repository (dark blue). Annotations represent the mean $R^2_{\text{Holdout}}$ (the central dots), while the range of the whiskers represent the minimum and maximum $R^2_{\text{Holdout}}$ across folds. For sample sizes, kindly see Table S6.

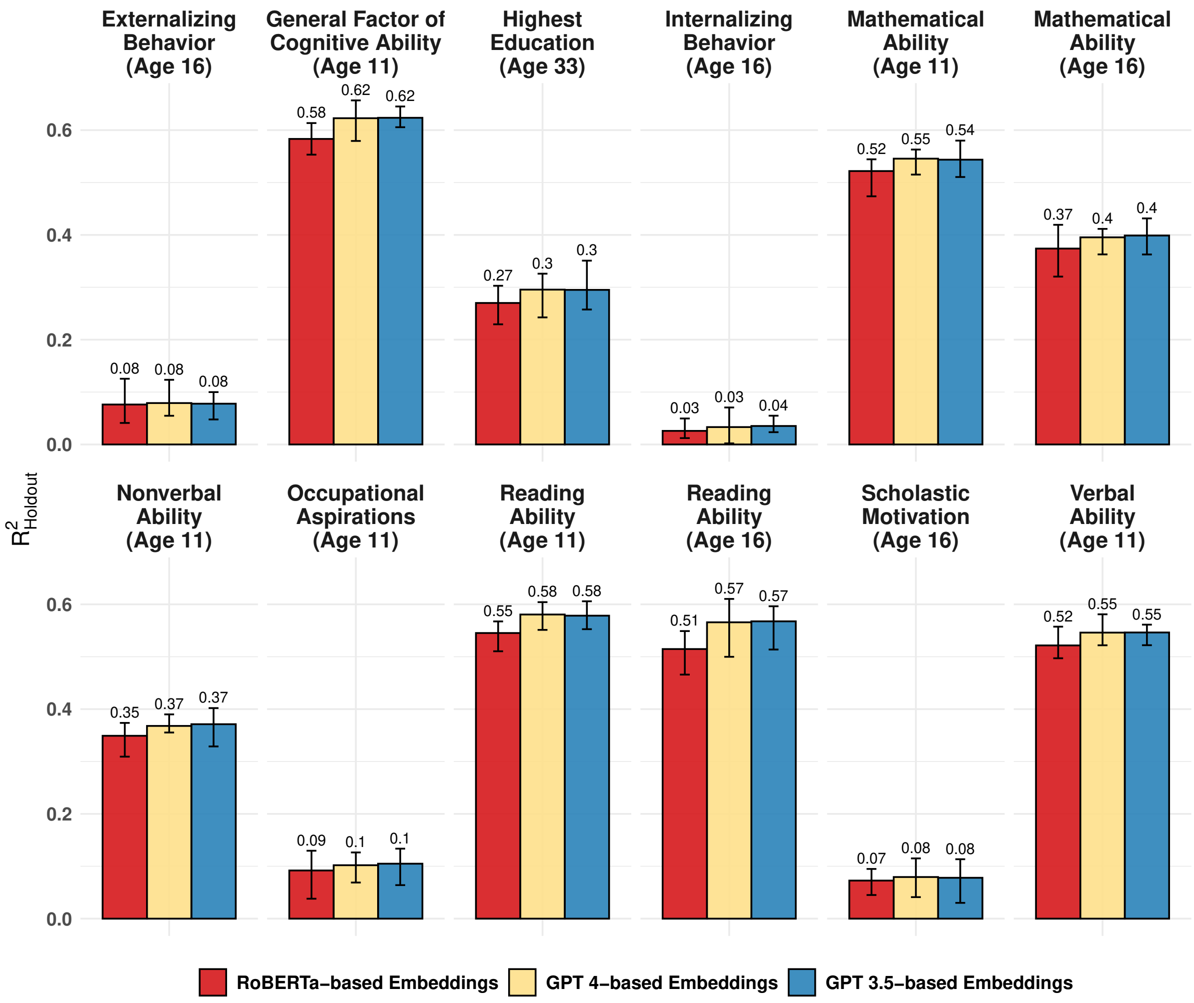


**Figure S2:** Comparison of different methods for extracting embeddings; RoBERTa, GPT-4, and GPT-3.5 (the method used in this article). Bar height represents the mean $R^2_{\text{Holdout}}$ value, while whiskers represent the minimum and maximum across all ten folds. For sample sizes, kindly see Table S6.

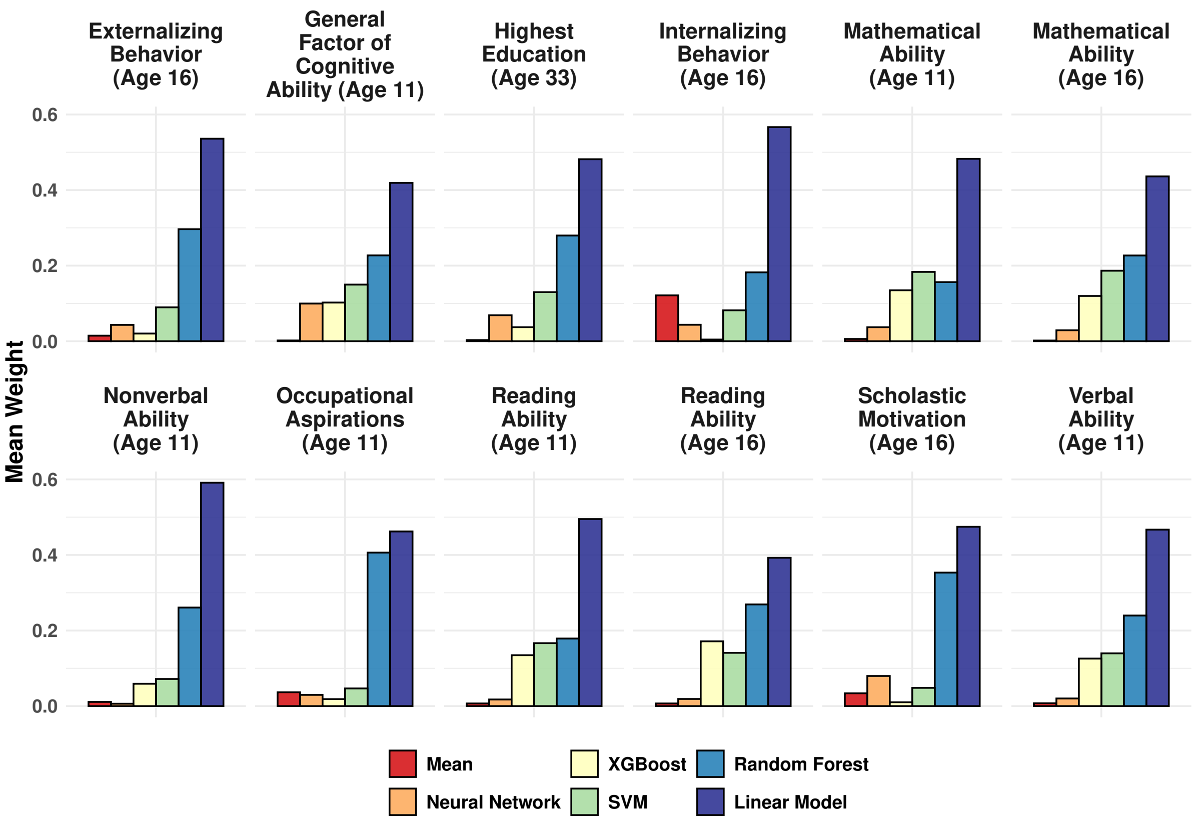


**Figure S3:** A visualisation of the weights attributed by the SuperLearner based approach to each of the composite models. The candidate models for potential inclusion and weigthing include the in-sample (training mean), a shallow neural network, eXtreme Gradient Boosting, a Support Vector Machine, a Random Forest, and a Generalised Linear Model. For sample sizes, kindly see Table S6.

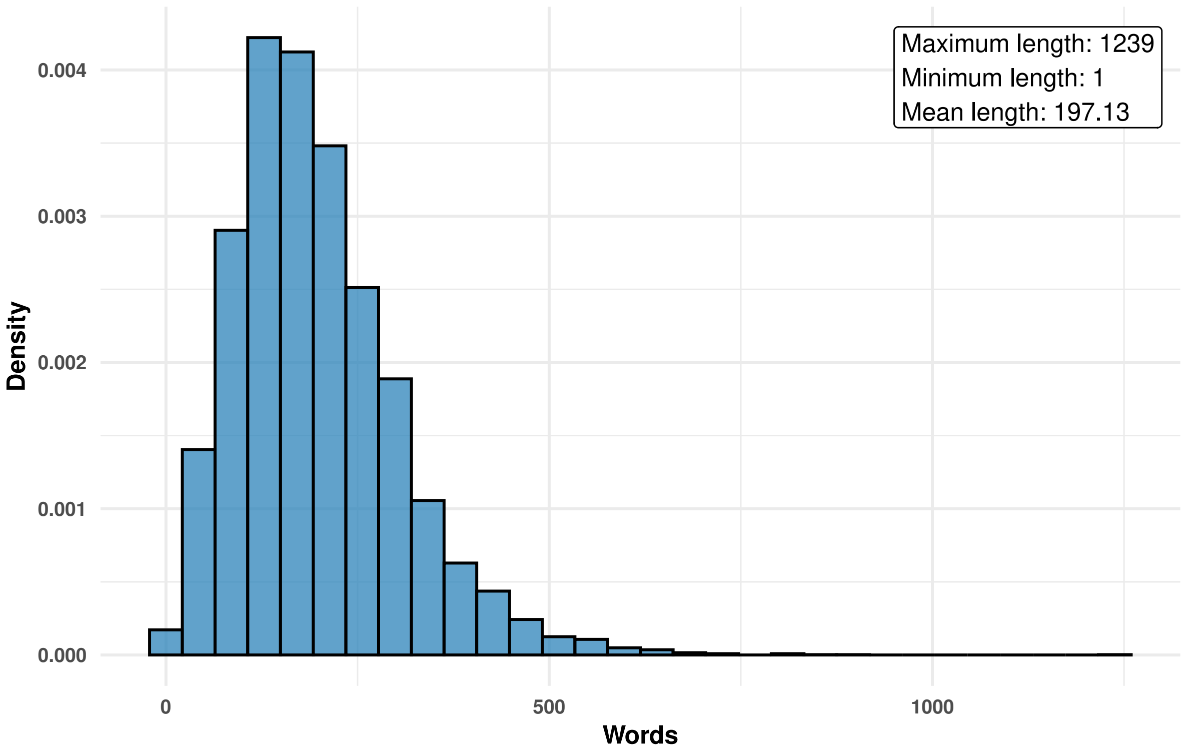


**Figure S4:** Length of Essays. The mean length of the essays (which were indicatively instructed to be 250 words) was 197.13 words, ranging from a minimum of one word, to a maximum of 1239 words. The standard deviation of the length is 106.23. Essays were manually transcribed. N = 10511.

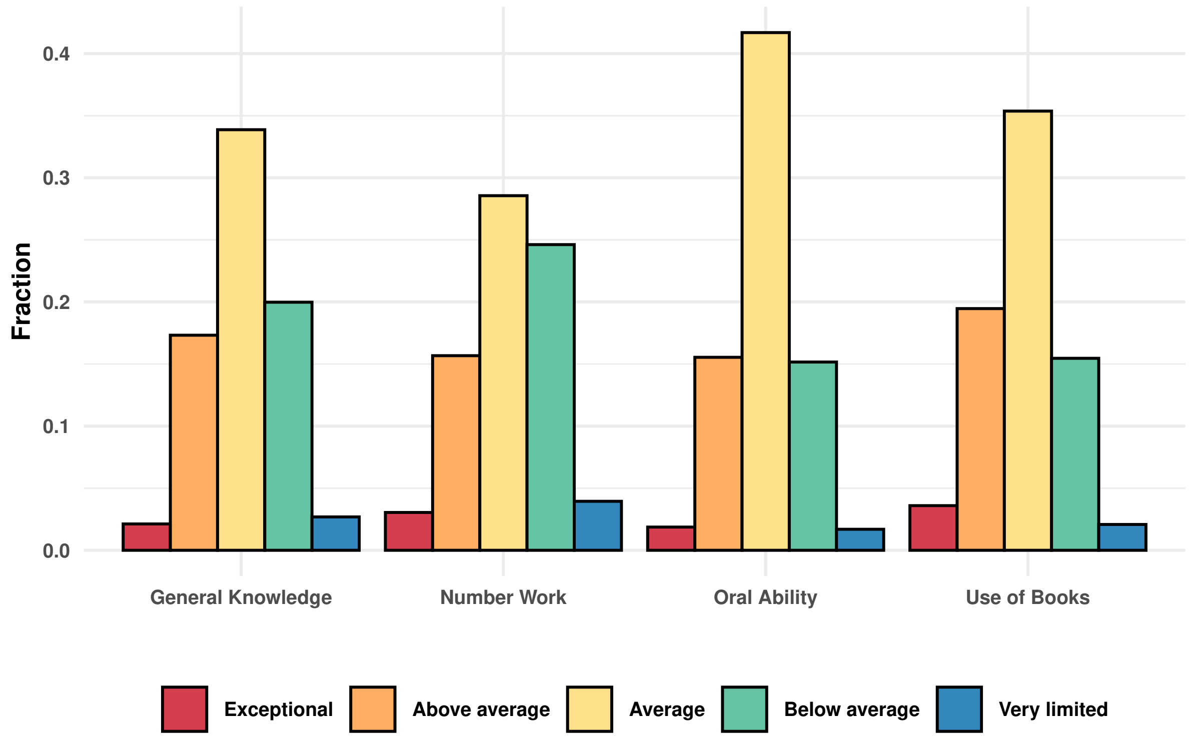


**Figure S5:** Distribution of Scholastic Teacher Assessments across four domains of ‘General Knowledge’, ‘Number Work’, ‘Oral Ability’, and ‘Use of Books’. Teachers could rate on a five point scale, ranging from ‘Very Limited’ to ‘Exceptional’. Source: NCDS data. N = 18558 (missings not shown).

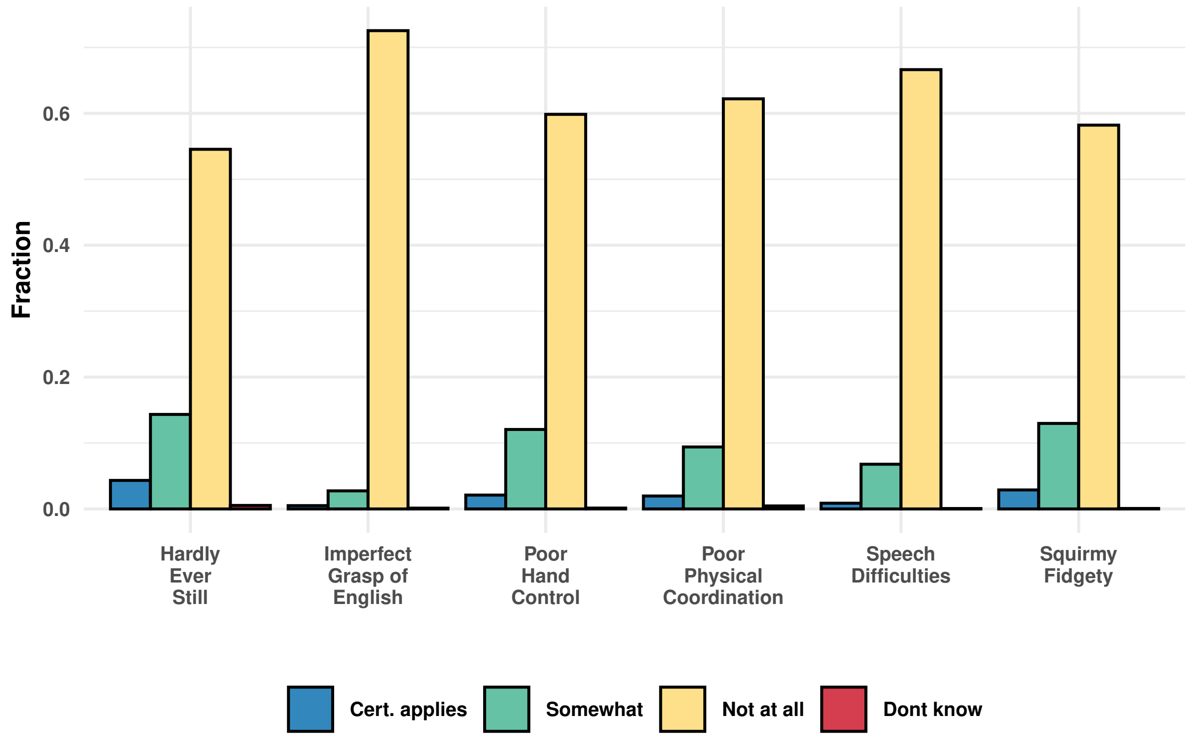


**Figure S6:** Distribution of Behavioral Teacher Assessments across six domains of 'Hardly Ever Still', 'Imperfect Grasp of English', 'Poor Hand Control', 'Poor Physical Condition', 'Speech Difficulties', and 'Squirmy/Fidgiety'. Teachers could rate on a four point scale, ranging from 'Certainly Applies' to 'Not at All'. Source: NCDS data. N = 18558 (missings not shown).

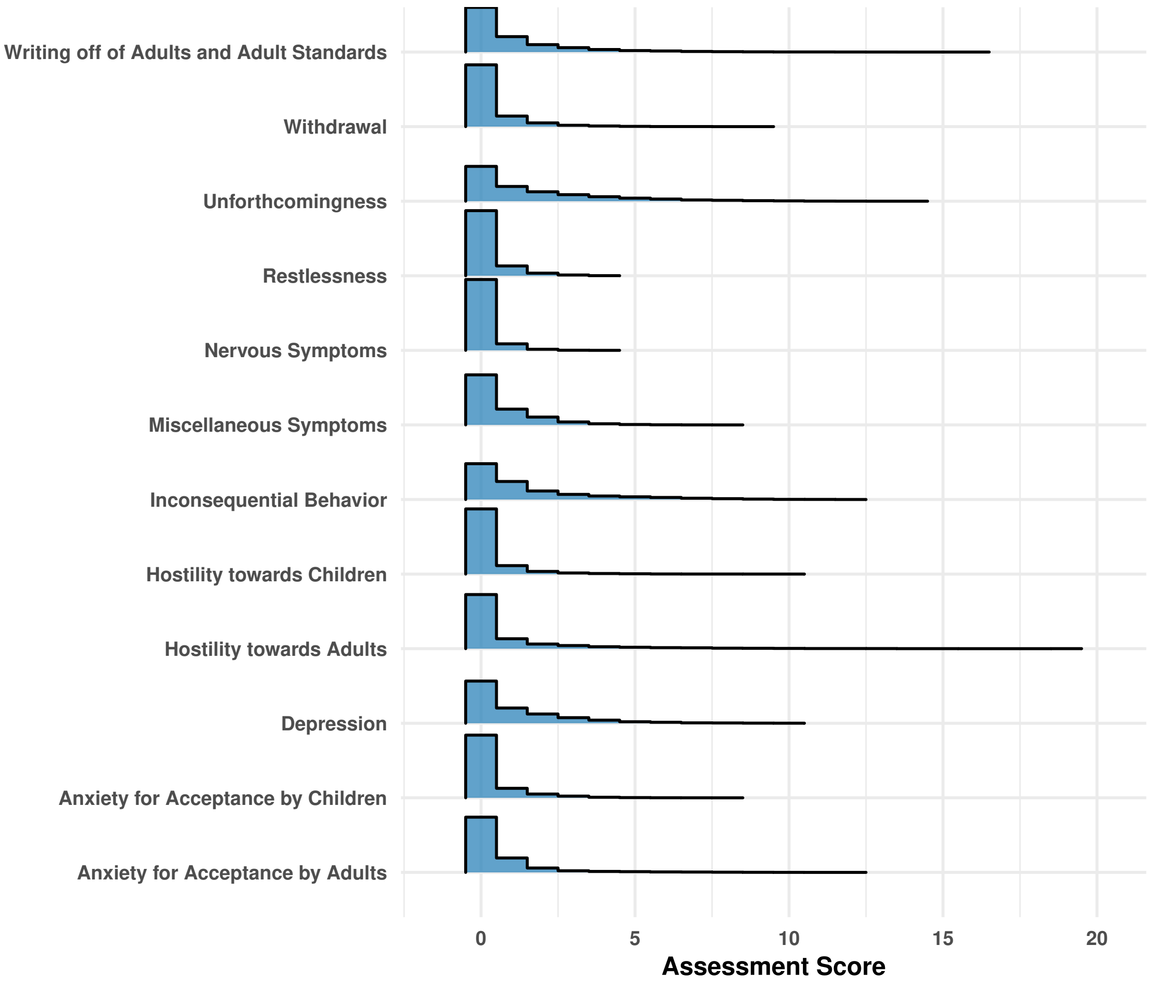


**Figure S7:** Distribution of Further Behavioral Teacher Assessments. Teachers were also asked to provide a quantitative assessment of further behavioural traits on an ordinal scale of 0-20. Source: NCDS data. N = 18558 (missings not shown).

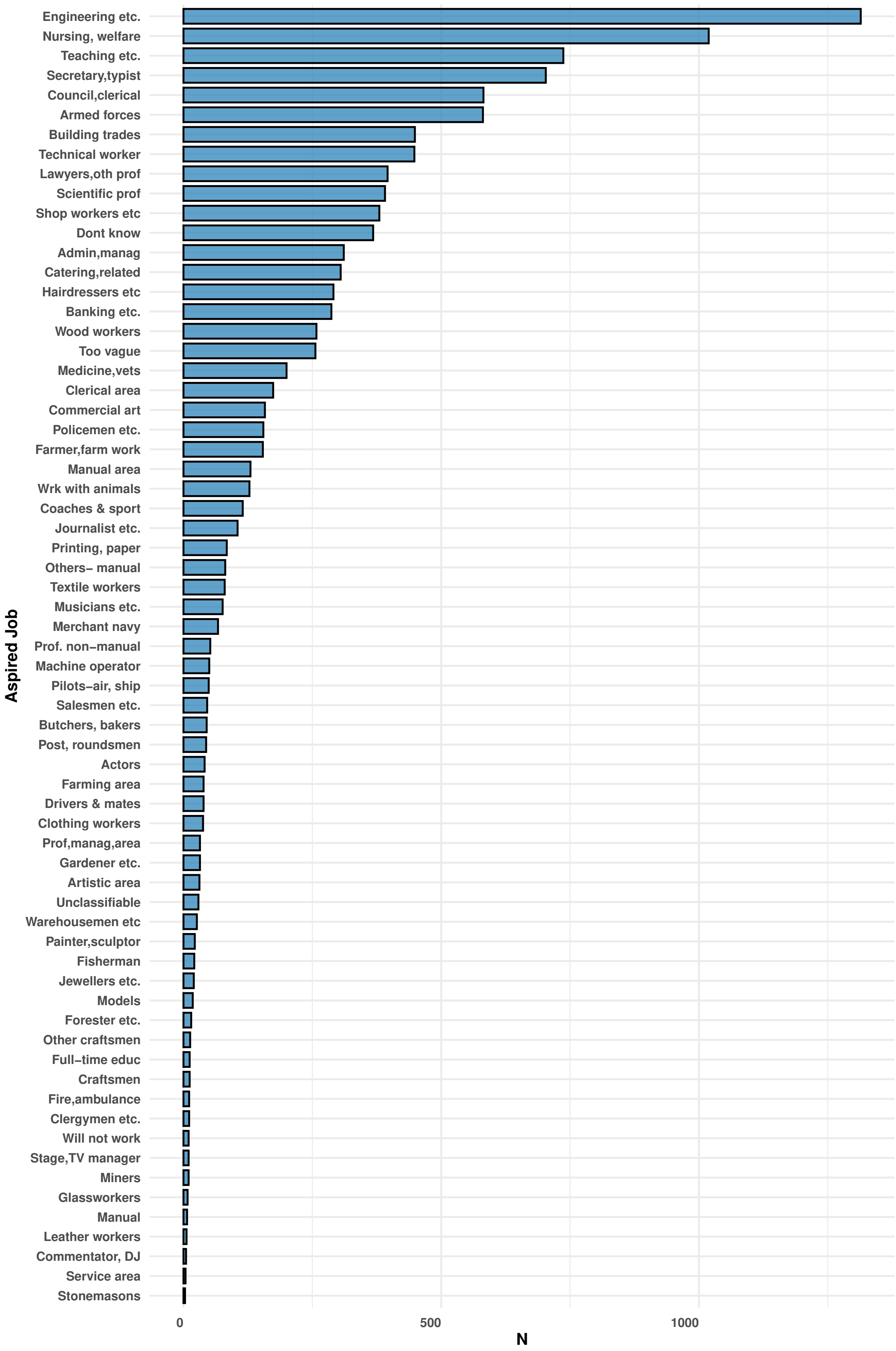


**Figure S8:** Distribution of Aspired Occupation. Members of the cohort study were asked – when they were 16 – which occupation they wanted to pursue as a career. N = 11594.

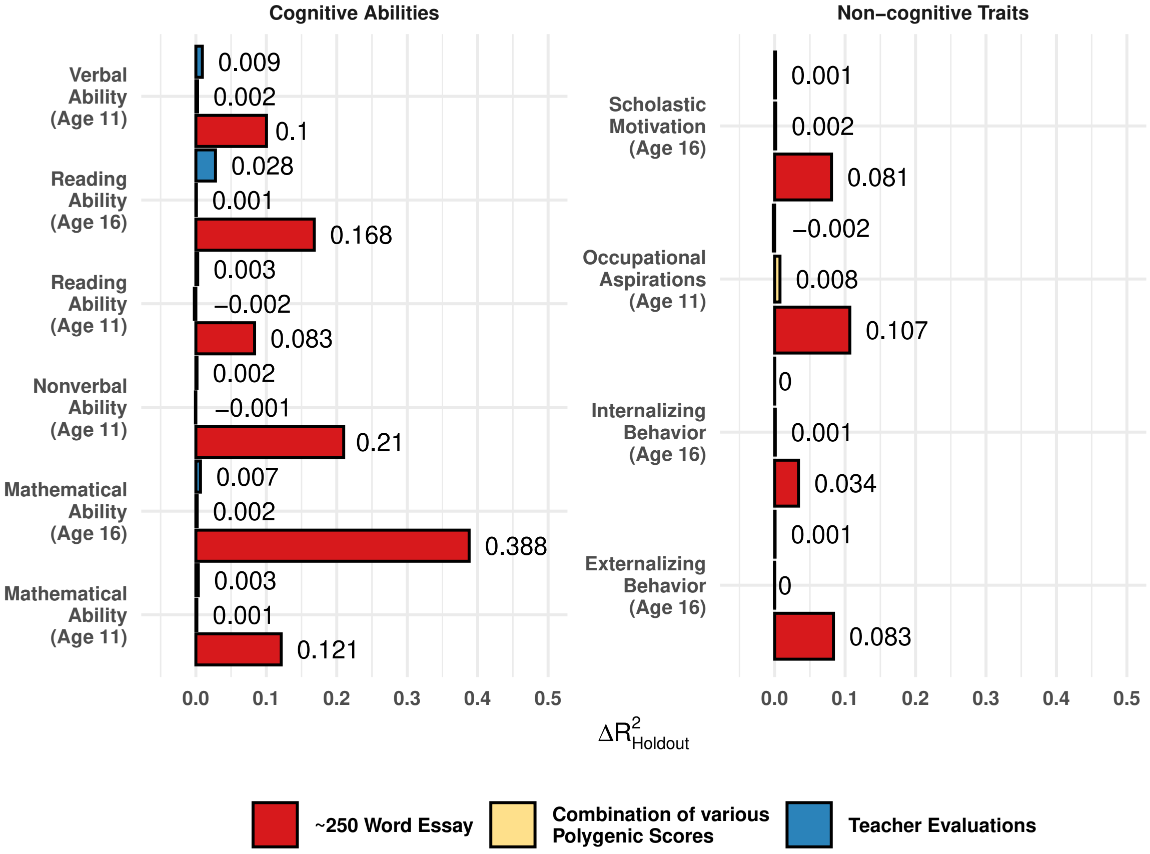


**Figure S9:** Incremental Improvement in Prediction. The figure visualises the gains in accuracy which can be atrributed to a SuperLearner when compared to a Linear Model. The results show – mostly – that the SuperLearner is able to consistently reduce 'Learning' or Estimation' error. For sample sizes, kindly see Table S6.

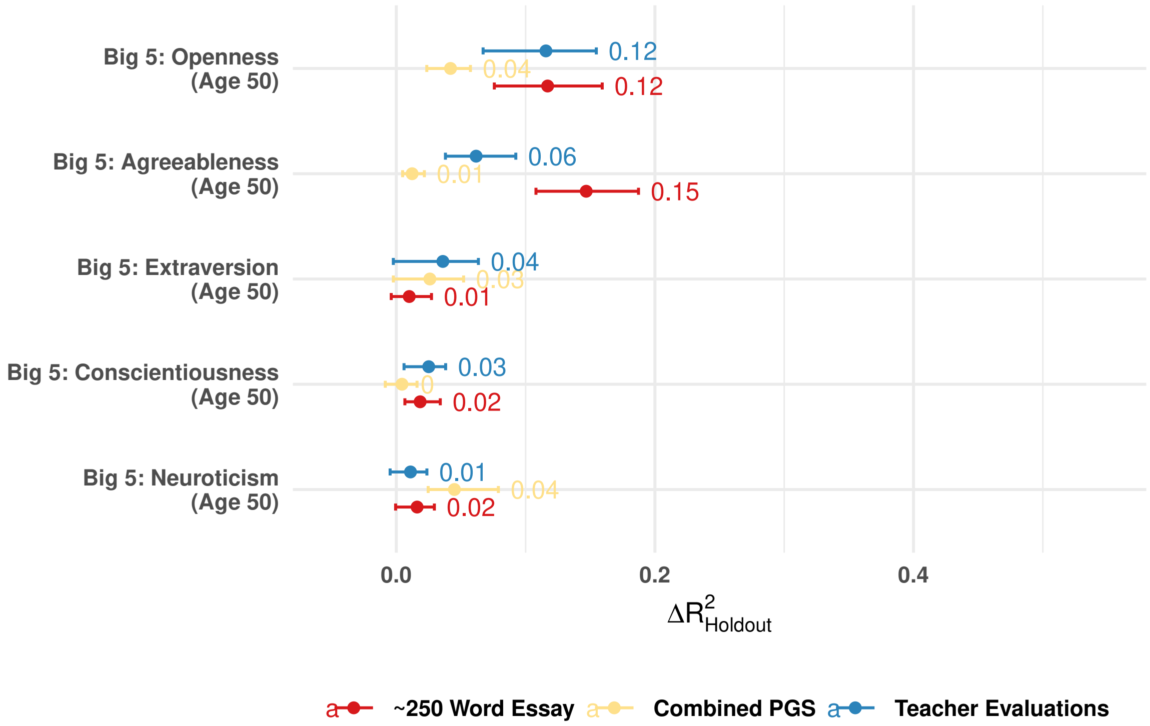


**Figure S10:** Prediction of Personality (Age 50). Sample sizes for Big 5 traits: Essay: E = 5538, A = 5520, C = 5450, N = 5522, O = 5468, Polygenic: E = 5077, A = 5073, C = 5014, N = 5081, O = 5027, Teacher: E = 6865, A = 6851, C = 6753, N = 6849, O = 6790. E=Extraversion, A=Agreeableness, C=Conscientiousness, N=Neuroticism, O=Openness.

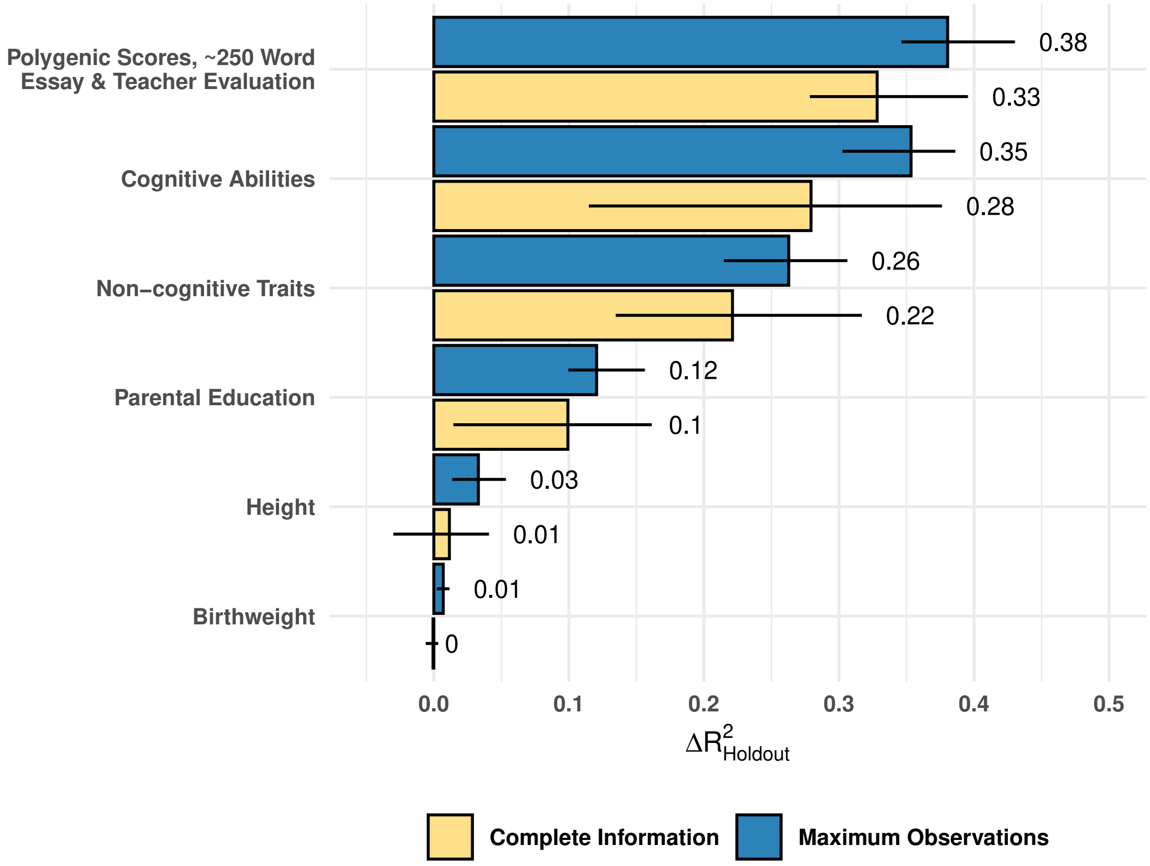


**Figure S11:** Comparison of Prediction for Maximum and Complete Samples (Educational Attainment). For sample sizes, kindly see Table S6.

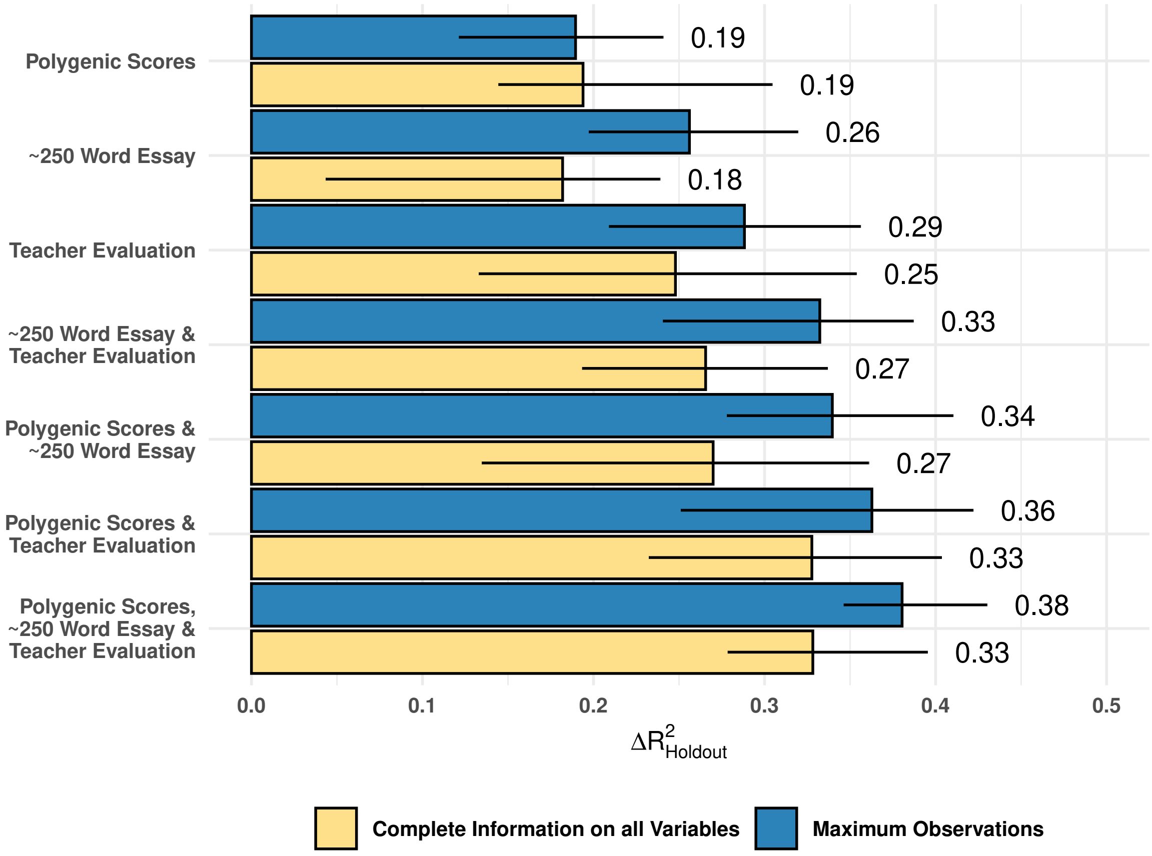


**Figure S12:** Comparison of Prediction for Maximum and Complete Samples (Educational Attainment using Teacher Assessments, Essays and Genetic Data). For sample sizes, kindly see Table S6.

# Supplementary Tables

**Table S1:** Sources of GWAS summary statistics used to build polygenic scores that were used as input to the SuperLearner.

| Category | Trait | Source |
|---|---|---|
| Cognition | Cognitive Performance | (38) |
| Cognition | General Factor of Cognitive Ability | (6) |
| Disease | ADHD | (8) |
| Disease | Autism Spectrum Disorders | (10) |
| Disease | Bipolar | (20) |
| Disease | Schizophrenia | (22) |
| Disease | Depression | (13) |
| Personality | Agreeableness | (19) |
| Personality | Anxiety and tension factors | (12) |
| Personality | Conscientiousness | (19) |
| Personality | Extraversion | (19) |
| Personality | Frequency of tenseness or restlessness in last 2 weeks | (26) |
| Personality | Neuroticism | (12) |
| Personality | non-cognitive Skills | (7) |
| Personality | Openness | (19) |
| Personality | Positive Affect | (42) |
| Personality | Risk Tolerance | (15) |
| Personality | Worry and vulnerability factors | (12) |
| Social | Age at First Birth | (1) |
| Social | Educational Attainment (excl. NCDS) | (38) |
| Social | Family relationship satisfaction | (26) |
| Social | Friendships satisfaction | (26) |
| Social | Household Income | (11) |
| Social | Life Satisfaction | (2) |
| Social | Social Activities: Pub or social club | (5) |
| Social | Social Activities: Religious group | (5) |
| Social | Social Activities: Sports club or gym | (5) |
| Substance | Age of smoking initiation | (18) |
| Substance | Alcohol consumption Drinks per week | (18) |
| Substance | Alcohol use (AUDIT-Score) | (24) |
| Substance | Cannabis Use Disorder | (14) |
| Substance | Cigarettes smoked per day | (18) |
| Substance | Smoking cessation | (18) |

**Table S2:** In-sample R-squared measures for traits and ten principal components with ordinary least squares

| **Outcome** | **R-squared** | **Adj. R-squared** | **P-value** | **Nobs** |
|---|---|---|---|---|
| General Factor of Cognitive Ability (Age 11) | 0.00241 | 0.000628 | 0.196 | 5614 |
| Verbal Ability (Age 11) | 0.00173 | -0.000522 | 0.467 | 5617 |
| Nonverbal Ability (Age 11) | 0.00310 | -0.000132 | 0.0658 | 5617 |
| Reading Ability (Age 11) | 0.00144 | -0.000344 | 0.622 | 5615 |
| Mathematical Ability (Age 11) | 0.00311 | 0.00133 | 0.0650 | 5615 |
| Reading Ability (Age 16) | 0.00274 | -0.000996 | 0.190 | 5007 |
| Mathematical Ability (Age 16) | 0.00272 | -0.000719 | 0.195 | 4985 |
| Occupational Aspirations (Age 11) | 0.00422 | 0.00227 | 0.0133 | 4563 |
| Scholastic Motivation (Age 16) | 0.00191 | -0.00135 | 0.500 | 4937 |
| Externalizing Behavior (Age 16) | 0.000974 | -0.00104 | 0.489 | 4873 |
| Internalizing Behavior (Age 16) | 0.00183 | -0.00158 | 0.513 | 5039 |
| Highest Education (Age 33) | 0.00241 | 0.000631 | 0.195 | 5619 |

**Table S3:** Out-of-sample R-squared measures for traits within a SuperLearner framework with 10 genetic principal components as predictors

| **Outcome** | **Mean R-squared (Min, Max)** |
|---|---|
| General Factor of Cognitive Ability (Age 11) | -0.0002 (-0.0071, 0.0053) |
| Verbal Ability (Age 11) | -0.0001 (-0.0025, 0.0028) |
| Nonverbal Ability (Age 11) | -0.0002 (-0.0022, 0.0007) |
| Reading Ability (Age 11) | -0.0001 (-0.0014, 0.0017) |
| Mathematical Ability (Age 11) | -0.0017 (-0.0216, 0.0064) |
| Reading Ability (Age 16) | 0.0018 (-0.0017, 0.0074) |
| Mathematical Ability (Age 16) | -0.0013 (-0.0045, 0.0008) |
| Occupational Aspirations (Age 11) | -0.0013 (-0.0124, 0.0012) |
| Scholastic Motivation (Age 16) | -0.0018 (-0.0070, 0.0017) |
| Externalizing Behavior (Age 16) | -0.0659 (-0.1197, -0.0017) |
| Internalizing Behavior (Age 16) | -0.0178 (-0.0731, 0.0069) |
| Highest Education (Age 33) | -0.0013 (-0.0132, 0.0030) |

**Table S4:** Out-of-sample R-squared measures for traits within a SuperLearner framework with 10 genetic principal components and the full set of polygenic scores as predictors

| **Outcome** | **Mean R-squared (Min, Max)** |
|---|---|
| General Factor of Cognitive Ability (Age 11) | 0.1666 (0.1255, 0.1943) |
| Verbal Ability (Age 11) | 0.1264 (0.0734, 0.1528) |
| Nonverbal Ability (Age 11) | 0.1107 (0.0787, 0.1470) |
| Reading Ability (Age 11) | 0.1417 (0.0890, 0.1720) |
| Mathematical Ability (Age 11) | 0.1641 (0.1136, 0.2021) |
| Reading Ability (Age 16) | 0.1452 (0.0761, 0.1784) |
| Mathematical Ability (Age 16) | 0.1701 (0.1125, 0.2059) |
| Occupational Aspirations (Age 11) | 0.0420 (0.0008, 0.0765) |
| Scholastic Motivation (Age 16) | 0.0534 (0.0309, 0.0807) |
| Externalizing Behavior (Age 16) | 0.0390 (0.0104, 0.0581) |
| Internalizing Behavior (Age 16) | 0.0068 (-0.0114, 0.0285) |
| Highest Education (Age 33) | 0.1942 (0.1329, 0.2502) |

**Table S5:** Sample Sizes for Main Figure 2.

| | Essay | PGSs | Evaluations |
|---|---|---|---|
| Verbal Ability (Age 11) | 10,489 | 5,617 | 12,957 |
| Nonverbal Ability (Age 11) | 10,489 | 5,617 | 12,957 |
| Reading Ability (Age 11) | 10,490 | 5,615 | 12,956 |
| Mathematical Ability (Age 11) | 10,489 | 5,615 | 12,953 |
| Occupational Aspirations (Age 11) | 7,040 | 4,563 | 8,701 |
| Factor of Scholastic Motivation (Age 16) | 7,509 | 4,897 | 9,295 |
| Externalizing Behavior (Age 16) | 7,734 | 4,973 | 9,598 |
| Internalizing Behavior (Age 16) | 7,874 | 5,039 | 9,753 |

**Table S6:** Sample Sizes for Main Figure 5.

| Predictor | N |
|---|---|
| Cognitive Abilities | 9,590 |
| Non-cognitive Traits | 7,061 |
| Birthweight | 10,547 |
| Height | 8,822 |
| Parental Education | 7,866 |
| Polygenic Scores, ~250 Word Essay & Teacher Evaluation | 3,399 |

**Table S7:** Loadings of cognitive ability measures (and variable IDs in NCDS) on general factor of cognitive ability in exploratory factor analysis. The general factor represents the general factor of cognitive ability predicted in the various models of the paper.

| | Loading | Communality | Uniqueness |
|---|---|---|---|
| Verbal score on general ability test (n914) | 0.928 | 0.862 | 0.138 |
| Non verbal score on gen ability test (n917) | 0.84 | 0.705 | 0.295 |
| Reading comprehension test score (n923) | 0.813 | 0.661 | 0.339 |
| Mathematics test score (n926) | 0.882 | 0.777 | 0.223 |
| Proportion of Variance Explained | 0.751 | | |

**Table S8:** Loadings of scholastic motivation measures (and variable IDs in NCDS) on general factor of scholastic motivation in exploratory factor analysis. The general factor represents the scholastic motivation behaviour variable predicted in the various models of the paper.

| | Loading | Communality | Uniqueness |
|---|---|---|---|
| I do not like school (n2721) | 0.822 | 0.676 | 0.324 |
| School waste of time (n2716) | 0.759 | 0.576 | 0.424 |
| Homework is a bore (n2718) | 0.673 | 0.452 | 0.548 |
| I never take work seriously (n2720) | 0.667 | 0.444 | 0.556 |
| Proportion of Variance Explained | 0.537 | | |

**Table S9:** Loadings of externalizing behaviour measures (and variable IDs in NCDS) on general factor of externalizing behaviour in exploratory factor analysis. The general factor represents the externalizing behaviour variable predicted in the various models of the paper.

| Outcome | Loading | Communality | Uniqueness |
|---|---|---|---|
| Is often disobedient (n2310) | 0.925 | 0.856 | 0.144 |
| Frequently fights, very quarrelsome (n2300) | 0.898 | 0.806 | 0.194 |
| Often tells lies (n2314) | 0.888 | 0.788 | 0.212 |
| Destroys, damages own, others property (n2299) | 0.883 | 0.779 | 0.221 |
| Bullies other children (n2321) | 0.882 | 0.777 | 0.223 |
| Resentful, aggressive when corrected (n2320) | 0.861 | 0.741 | 0.259 |
| Restless, difficulty staying seated (n2296) | 0.826 | 0.683 | 0.317 |
| Irritable, touchy, flies off the handle (n230) | 0.821 | 0.675 | 0.325 |
| Cannot settle more than a few moments (n2311) | 0.813 | 0.661 | 0.339 |
| Squirmy, fidgety (n2298) | 0.789 | 0.622 | 0.378 |
| Has stolen at least once in past year (n2315) | 0.749 | 0.561 | 0.439 |
| Proportion of Variance Explained | 0.723 | | |

**Table S10:** Loadings of internalizing behaviour measures (and variable IDs in NCDS) on general factor of internalizing behaviour in exploratory factor analysis. The general factor represents the internalizing behaviour variable predicted in the various models of the paper.

| Outcome | Loading | Communality | Uniqueness |
|---|---|---|---|
| Often appears miserable, unhappy, etc (n2305) | 0.851 | 0.725 | 0.275 |
| Tears on arrival, refusal to enter school (n2318) | 0.742 | 0.551 | 0.449 |
| Often worries about many things (n2302) | 0.728 | 0.53 | 0.47 |
| Fearful of new situations & things (n2312) | 0.703 | 0.494 | 0.506 |
| Tends to be on own (n2303) | 0.634 | 0.402 | 0.598 |
| Proportion of Variance Explained | 0.54 | | |

**Table S11:** Best Model Predictions of Big Five Measures.

| Outcome | NCDS Variable |
|---|---|
| IPIP Personality Inventory - Extraversion score 5-50 | nd8ext |
| IPIP Personality Inventory - Agreeableness score 5-50 | nd8agr |
| IPIP Personality Inventory - Conscientiousness score 5-50 | nd8con |
| IPIP Personality Inventory - Emotional Stability score 5-50 | nd8emo |
| IPIP Personality Inventory - Intellect Score 5-50 | nd8int |

**Table S12:** Sample Size for each Combination of Variables used .

| | Teacher Assessments | Essays | Genetic Data | Teacher, Essays, PGS | All Variables |
|---|---|---|---|---|---|
| General Factor of Cognitive Ability (Age 11) | 12,951 | 10,488 | 5,614 | 3,838 | 1,618 |
| Verbal Ability (Age 11) | 12,957 | 10,489 | 5,617 | 3,838 | 1,618 |
| Nonverbal Ability (Age 11) | 12,957 | 10,489 | 5,617 | 3,838 | 1,618 |
| Reading Ability (Age 11) | 12,956 | 10,490 | 5,615 | 3,838 | 1,618 |
| Mathematical Ability (Age 11) | 12,953 | 10,489 | 5,615 | 3,838 | 1,618 |
| CAMSIS-Prestige of first aspired Job (Age 11) | 8,701 | 7,040 | 4,563 | 2,795 | 1,618 |
| Factor of Scholastic Motivation (Age 16) | 9,295 | 7,509 | 4,897 | 2,995 | 1,618 |
| Externalizing Behavior (Age 16) | 9,598 | 7,734 | 4,973 | 3,033 | 1,618 |
| Internalizing Behavior (Age 16) | 9,753 | 7,874 | 5,039 | 3,073 | 1,618 |
| Highest Education (Age 33) | 8,892 | 7,190 | 5,619 | 3,399 | 1,618 |
